# Germany's Data Transparency Regulations – potential, developments and RKI federal health reporting activities

**DOI:** 10.17886/RKI-GBE-2018-056

**Published:** 2018-05-16

**Authors:** Alexander Rommel, Christian Schmidt

**Affiliations:** Robert Koch Institute, Berlin, Department of Epidemiology and Health Monitoring

Since 2014, claims data of statutory health insurance companies in Germany, which provides a basis to calculate the morbidity-oriented risk structure compensation scheme (Morbi-RSA), can be analysed by certain institutions for defined purposes. Book 5 of the German Social Code sections 303a et seq. as well as Germany’s Data Transparency Regulations (DaTraV) sets the details out more clearly. DaTraV applies to the data of all statutorily insured persons in Germany, and access to this data can be applied for at the German Institute of Medical Documentation and Information (DIMDI) for specific questions. The data set contains information on outpatient and inpatient care, costs and prescribed medications. Potentially this data could allow estimates on prevalence and incidence rates based on the data of roughly 70 million statutorily insured. One limitation is the scope of this data, as it only includes part of all claims data (excluding for example data on long-term care insurance). Moreover, it currently takes four years from data collection to data provision, after which those wishing to access the data still need to go through a lengthy application process. After the first phase of incoming data applications the procedures have been internally evaluated resulting in a current revision of DaTraV with regard to making access to data easier and speeding up the application process. An initial analysis in the context of diabetes surveillance at the Robert Koch Institute indicates that roughly one in seven publicly insured patients aged over 40 has been diagnosed with diabetes. Clearly, the combination of in and outpatient data provides benefits to estimating prevalence and incidence rates. Compared to the results from outpatient billing data of Germany’s Associations of Statutory Health Insurance Physicians, the analysis of DaTraV data shows higher estimates of diabetes prevalence because the data provide a more realistic picture of actual health care provision. By enabling population-wide estimates on disease frequency, DaTraV data importantly contributes to the further development of health reporting. Federal health reporting (GBE) at the Robert Koch Institute is involved in the permanent development of this information system. It actively participates in the health care data provision working group of Germany’s umbrella organisation for networked medical research TMF e.V. Moreover, in co-operation with DIMDI, it develops standard analyses that represent a key element in the establishment of a routine provision of DaTraV data for health reporting and surveillance.

